# Change of Electronic Structures by Dopant-Induced Local Strain

**DOI:** 10.1038/srep11227

**Published:** 2015-06-10

**Authors:** Gyu Hyeong Kim, Sukmin Jeong

**Affiliations:** 1 Department of Physics and Research Institute of Physics and Chemistry, Chonbuk National University, Jeonju 561-756, Korea

## Abstract

Ag-induced Si(111)-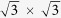 surfaces (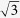-Ag) exhibit unusual electronic structures that cannot be explained by the conventional rigid band model and charge transfer model. The (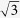-Ag surfaces feature a free-electron-like parabolic band, the *S*_1_ band, that *selectively* shifts downward upon the adsorption of noble metal or alkali metal adatoms. Furthermore, the downward shift of *S*_1_ is *independent* of the type of dopants, Au, Ag, and Na. According to charge transfer analysis, Au adatoms accumulate electrons from the substrate and become negatively charged, whereas Na adatoms become positively charged, which indicates that *S*_1_ should shift in the opposite direction for both the adatoms. Investigation of calculated structures, calculation of model structures, and tight-binding analysis disclose that the changes in the electronic structure are closely related to the average Ag-Ag distance in the substrate and have their origin in the local strain induced by dopants (adatoms). This explanation implies that the electronic structure is irrespective of the dopant characters itself and paves a new way for understanding the electronic structures associated with the presence of dopants.

The electronic structure of a material can undergo changes in the presence of foreign atoms (dopants). These changes form the basis of a fundamental research topic in the field of condensed matter physics. Electronic structures that may be tuned through control over the type or quantity of dopant present in the material feature in a wide range of practical applications. Current semiconductor device designs exploit these effects by introducing heterogeneous impurities that act as electron donors or acceptors with respect to the semiconductor. The electronic structure of graphene, including the band gap and the polarity change, may be modified using electron or hole doping. These methods are currently the subject of intensive investigations that seek to improve the utility of graphene in a variety of practical applications. The rigid band model, in which the electronic structure is understood in terms of the host material’s electronic structure^1^, offers a broadly useful model of doping. The dopant donates or accepts charge to or from the host material. The electronic structures of doped topological insulators^2^,^3^ or graphene^4^ may be understood in terms of this framework.

The electronic structure of Ag-induced Si(111)-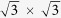 (hereafter, 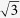-Ag) surfaces is strange in this sense. The 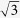-Ag surfaces act as a two-dimensional electron gas (2DEG) system in which the surface bands (*S*_1_) are characterized by a parabolic dispersion that creates the free electron gas conditions^5^,^6^,^7^,^8^,^9^,^10^,^11^,^12^,^13^. The introduction of a dopant (adatom, in this case), such as an alkali or noble metal, shifts only the *S*_1_ band downward in proportion to the dopant coverage. These selective shifts of *S*_1_ are inconsistent with the standard rigid band model. Furthermore, an interesting feature of this system is that the electronic structures observed using angle-resolved photoemission spectroscopy (ARPES) are nearly equivalent in the context of doping with a variety of adatoms: Na, Ag, or Au^10^. These band shifts have been ascribed to charge transfer from the dopant to the surface band^6^,^7^,^8^,^9^,^10^,^11^,^12^; however, the electron affinities of the elements, 1.39 eV for Si, 1.30 eV for Ag, 2.31 eV for Au, and 0.55 eV for Na^14^,^15^,^16^,^17^, indicate that Au functions as an acceptor whereas Na functions as a donor. That is, the *S*_1_ band should shift in the opposite direction upon Au or Na adatom doping. Clearly, a traditional charge transfer model fails in the present system; thus a new mechanism must be proposed to explain the changes in the electronic structures of the 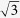-Ag surfaces in the presence of adatoms.

In this report we investigate the anomalous band shift observed in systems comprising 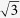-Ag surfaces doped with Au, Na, or Ag adatoms using first-principles calculations for the atomic and electronic structures obtained under a variety of coverage levels. The charge analysis described here reveals that charge transfer occurs from the substrate to the adatom in the context of Au doping, and the direction of charge transfer is reversed in the context of Na doping, as expected based on the electron affinities. Despite the differences in the charge polarities of the adatoms relative to the substrate, the electronic structures calculated for a given adatom coverage are similar for Au, Na, and Ag adatom doping, in agreement with the ARPES data. Furthermore, the downward shift in *S*_1_ increases in magnitude with the adatom coverage. We find that the magnitude of the band shift is correlated with the average distance between the surface Ag atoms. A plot of the calculated band shift as a function of the adatom height and a simple tight-binding argument support this model.

## Results

**Figure 1:**
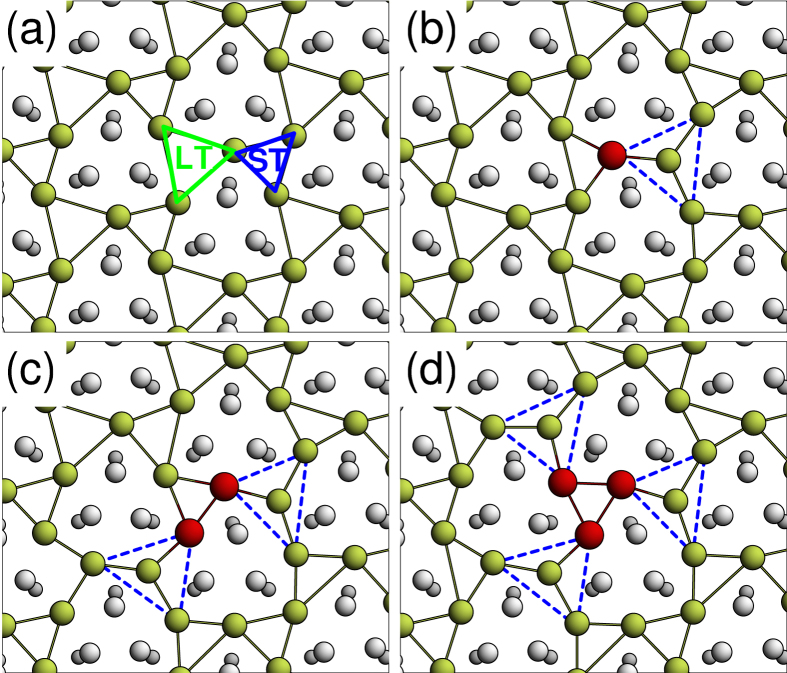
Calculated atomic structures of (**a**) the 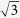-Ag substrate and substrates prepared with (**b**) one Au (0.021 ML), (**c**) two Au (0.042 ML), or (**d**) three Au adatoms (0.063 ML), respectively. White spheres represent the substrate Si atoms that formed Si triangles (SiTs), and the yellow and red spheres represent substrate Ag atoms and Au adatoms, respectively. The green and blue solid lines in (**a**) indicate the large triangle (LT) and the small triangle (ST) in the IET model, respectively. The dashed triangles in (**b**)–(**d**) represent the STs expanded by the exchanged Ag atoms.

The atomic structure of the 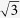-Ag surface is appropriately modeled using the inequivalent triangle (IET) model^18^, in which the surface Ag atoms form two types of inequivalent triangles: small triangles (ST) and large triangles (LT), and the Si atoms formed Si trimer (SiT) surrounded by six Ag triangles, as shown in Fig. 1(a). An Ag adatom occupies on a ST site which is the most stable, and its incorporation into the substrate is accompanied by considerable changes in the bond network around the adatom^19^. On the other hand, according to our recent paper^20^, the Au adatom that has deposited at the LT site easily exchanges the position with the neighboring Ag atom occupying nearby ST site, as indicated by the dashed triangle in Fig. 1(b). The structure shown in Fig. 1(b) is nearly equivalent to the structure obtained upon occupation of an ST site with a single Ag adatom. We find that a greater number of Au adatoms occupy stable positions upon the formation of an Au cluster, as shown in Fig. 1(c,d). The structures of the Ag^11^ and Na^21^ adatom clusters differ slightly from those shown in Fig. 1.

The calculated adsorption energies, defined as the energy gain of the cluster structures with respect to the energies of the substrate and free atoms, are 3.25, 3.34, and 3.58 eV/Au for one, two, and three Au adatoms, respectively. These values exceed the values obtained from Ag adatoms (~2.50 eV/Ag) or Na adatoms (~1.81 eV/Na). The higher adsorption energies of the Au adatoms are a consequence of the larger Au-Au, Au-Ag, and Au-Si bond energies compared with the Ag-Ag and Ag-Si bond energies. The bond energies of Au-Au, Au-Ag, Ag-Ag, Au-Si, and Ag-Si are calculated as 2.29, 2.20, 1.79, 3.70 and 2.80 eV, respectively, for the Au-Au, Au-Ag, Ag-Ag dimer, and (Ag, Au)-SiH_3_ complex structures. The Au-Au, Ag-Au, and Au-Si bond lengths of the most stable structures prepared using three Au adatoms are 2.95, 2.80, and 2.44–2.65 Å, respectively, that is, comparable to the bond lengths of the three Ag adatoms^11^.

The Bader analysis is used to calculate the magnitude of charge transferred from the Au, Ag, and Na adatoms to the substrate (see Methods). The calculation results are summarized in Table 1. The Au adatom extracts a 0.45*e* electronic charge from the surface (Ag layer + Si trimer) and bulk Si atoms, mainly from the Si trimers, whereas the Na adatom donates a charge of 0.80*e* to the surface Ag atoms and Si trimers. On the other hand, the Ag adatom remains almost neutral. These results indicate that the Au (Na) adatom functions as an electron acceptor (donor) on the 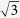-Ag surface, and the Ag adatom does not affect the charge state of the substrate. The adatom charge accumulation trend is consistent with the trend in the electron affinities. The magnitude of charge transfer increases with the adatom coverage, i.e., the magnitude of the charge transferred per adatom remains nearly constant.

**Figure 2:**
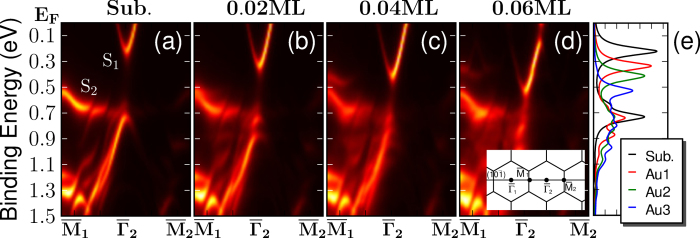
Simulated ARPES spectra for the (**a**) 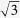-Ag substrate, (**b**) one, (**c**) two, or (**d**) three Au adatoms on the 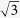-Ag substrate with 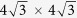 periodicity, corresponding to adatom coverages of 0.02, 0.04, and 0.06 ML, respectively. The inset in (**d**) displays the first and second surface Brillouin zone (SBZ) of the 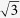-Ag substrate. The black, red, green, and blue lines in (**e**) indicate the intensity profiles of *S*_1_ at the 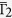 position for (**a**) 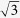-Ag, (**b**) one , (**c**) two, and (**d**) three Au adatoms, respectively.

We next simulate the ARPES spectra of the pristine 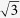-Ag surface and the surface in the presence of Au adatoms, as represented in the surface Brillouin zone (SBZ) of the 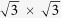 unit cell. During the ARPES spectra simulations, as shown in Fig. 2, the electronic bands in the SBZ of the super cell are unfolded into the SBZ of the 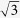-Ag using the method described in Methods. The calculated results can be compared directly with the experimental results^9^ by plotting the electronic bands around 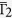, the center of the second SBZ. In the pristine 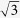-Ag case shown in Fig. 2(a), the simulated spectrum displays a characteristic free electron-like band near the Fermi level and a rather flat band near a binding energy of 0.7 eV. Both bands correspond to the experimentally observed *S*_1_ and *S*_2_ bands. In the present calculations, the *S*_1_ band is separated from the *S*_2_ band by 0.51 eV, which is smaller than the experimental value of ~0.75 eV. The *S*_1_ band mainly originates from the *p*_*x*_ and *p*_*y*_ orbitals of Ag and the *S*_2_ band corresponding to the Ag 5*s* orbital. The simulated spectrum obtained for the 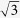-Ag substrate is consistent with the spectra calculated previously^11^ or derived from experiment^10^.

Figure 2(b–d) show the simulated ARPES spectra obtained from a surface in the presence of a single Au adatom, two Au adatoms, or three Au adatoms in the super cell, corresponding to 0.02, 0.04, or 0.06 ML, respectively. The parabolic *S*_1_ band shifts downward as the Au adatom coverage increases, in accordance with the experimental results^9^. The magnitudes of the band shift at 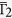 were 0.11, 0.19, and 0.31 eV for 0.02, 0.04, and 0.06 ML, respectively. Experimentally, the observed band shifts are ~0.16 and ~0.28 eV for 0.02 and 0.03 ML, respectively. Although the calculated values are smaller than the experimental values, the overall features agree well with experiments, considering the small band separation between *S*_1_ and *S*_2_ in the calculation.

The ARPES spectra of the Na and Ag adatoms (not shown) are nearly identical to those obtained in the presence of the Au adatoms, consistent with the experimental results. Despite the different charge polarities of the adatoms (positive Na, neutral Ag, and negative Au), the observation of similar *S*_1_ band shifts require a completely different interpretation. We note three effects that result from the presence of all kinds of the adatoms: (i) a downward band shift signifies a higher binding energy, (ii) the *S*_1_ band mainly originates from the Ag *p*_*x*_ and *p*_*y*_ orbitals, and (iii) the immersed adatom induces significant distortions in the local structure of the 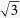-Ag surface. These three effects suggest that the band shift is strongly related to the local structure induced by the immersed adatoms, i.e., the Ag-Ag bonds in the substrate are strengthened.

**Figure 3:**
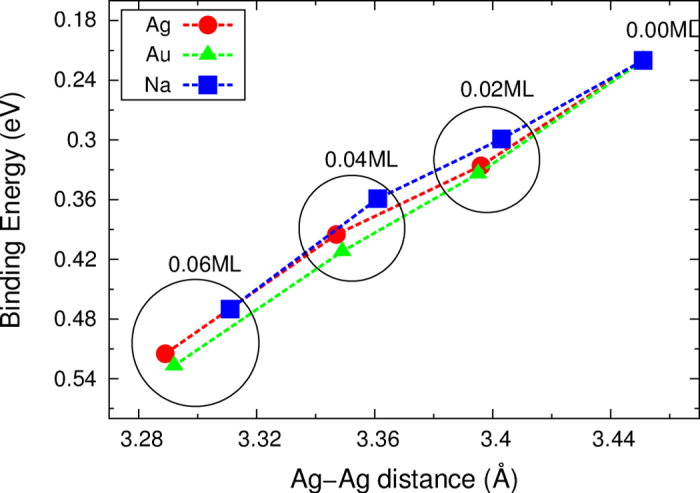
Binding energies of the *S*_1_ band minima at the 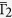 point as a function of the average Ag-Ag distance. The red circles, green triangles, and blue rectangles indicate the results obtained from the Ag, Au, and Na adatoms, respectively.

Because the bond strength is inversely proportional to the corresponding bond lengths, we investigate the shift in the *S*_1_ band as a function of the Ag-Ag bond length, as shown in Fig. 3. The average Ag-Ag distance (*d*_*avg*_) is calculated using the equation 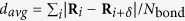, where the index *i* indicates the *i*-th Ag atoms other than the adatoms, *i* + *δ* represents the nearest neighboring Ag atom of the atom *i*, and *N*_bond_ indicates the total number of Ag-Ag bonds present in the calculated super cell. As shown in the figure, the magnitude of the band shift is approximately proportional to the decrease in the Ag-Ag distance, which is related to the adatom coverage. Thus, the adatom coverage and the band shift appear to be linearly related, as measured in the ARPES experiments.

## Discussion

**Figure 4:**
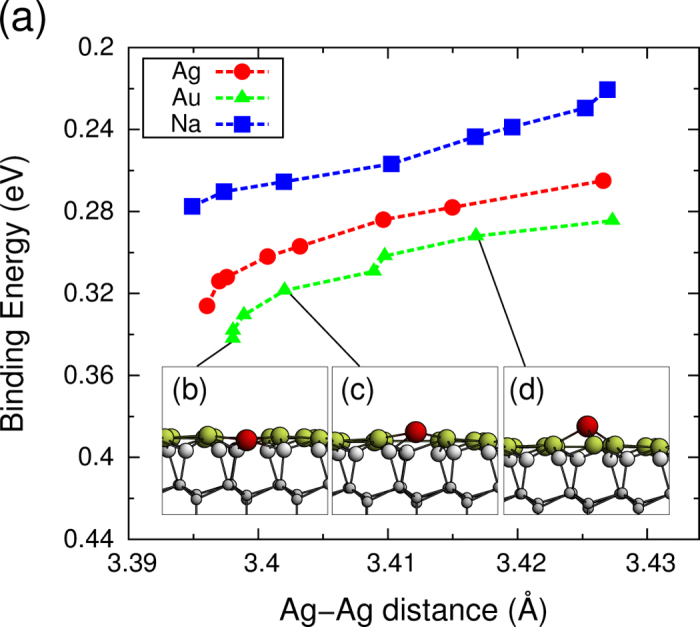
Binding energies of the *S*_1_ band minima at the 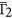 point as a function of the average Ag-Ag distance, which was controlled by the adatom height. The insets in (**b**–**d**) show the side views of the model structures in the presence of the Au adatoms at heights of 0.00, 0.75, and 1.50 Å from the surface, respectively.

The relationship between the adatom coverage and the magnitude of the band shift is further explored by performing calculations using a model system in which an adatom is present at a fixed height on the 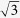-Ag surface. The adatoms are positioned at the ST site, and all coordinates except for the height are relaxed along the direction of force. The electronic structure of the model system is calculated for various heights and displays a shift in the *S*_1_ band for the Au, Ag, and Na adatoms, as shown in Fig. 4. The average Ag-Ag distance decreases as the adatom is incorporated into the surface level. Consequently, the *S*_1_ bands shifts downward in the presence of all types of adatom. The results obtained here reveal a very important property of the origin of the band shift. *Without* a change in the adatom coverage, the band could be shifted only through structural changes.

The physical origin of the selective band shift is investigated using a simple tight-binding model. The Ag overlayer is described using the Hamiltonian,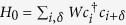, where the index *i* indicates the *i*-th lattice point and *i* + *δ* indicates the nearest neighbor of *i*. With 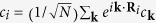, the eigenvector and eigenvalue of *H*_0_ are 
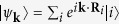
 and 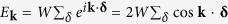, respectively. The adatom effects are observed by replacing a Ag atom at the *i*′ site with an adatom. Instead of changing the atomic structure, for simplicity, the bond strength around the atom *i*′ is enhanced, and the corresponding hopping parameter is increased to *W*′. The Hamiltonian could be written as 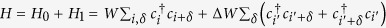, where Δ*W* = *W′*−*W*. The time-independent perturbation theory is applied: 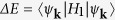, and the eigenvalue changes to 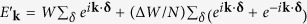. The eigenenergy could be rewritten as 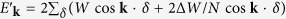, given the inversion symmetry. Because the minimum of *E*_*k*_ is −*W*, the energy minimum of the impurity system shifts downward to −*W* −2Δ*W*/*N* because Δ*W* > 0. The coefficient of the second term, 1/*N*, is related to the adatom coverage because in the present model, only one atom among the *N* atoms is designated an adatom. The last expression above indicates that the band shift resulting from the strengthened bond in the presence of the adatoms is proportional to the number of adatoms present. These features are consistent with the experimental observations.

The downward shift in the *S*_1_ band indicates an increase in the band occupancy. It could still be argued that because the main components of the *S*_1_ band are the substrate Ag orbitals, electronic charge is transferred from the adatoms to the substrate; however, it is nearly impossible to clearly distinguish between the substrate and the adatoms because the adsorbed adatoms induce structural rearrangements in the substrate. Thus, the adatoms and the substate are considered to form a single system, and the electronic charge of the whole system increases as the band shifts downward.

The Bader charge data in Table 1 shows that charge is mainly depleted from the Si trimers in the presence of Au adatoms and that the Si trimers and the Ag atoms obtains charge by almost equal amounts in the presence of Na adatoms. It is of note that the total charge of the whole surface layer (adatom + Si trimers + Ag atoms) is slightly negative and shows little variation according to the adatom. Although the surface bands other than *S*_1_ have their characteristic orbital features, the characteristics are weak since the surface bands are a mixture of the orbitals of many atoms. Thus the charge redistribution produced by the adatoms seems to have little effects on the surface bands except for *S*_1_.

In the present mechanism, the electronic structure can be changed through the lattice distortion induced by foreign atoms. This suggests that the mechanism has no restriction on the atomic species involved in the system neither the dimension in which the foreign atoms are located. Hence, we expect that the mechanism could be equally applied to a three-dimensional (3D) bulk crystal with interstitial impurities. What is important is how much the host material reacts with respect to the impurities. Further experimental and theoretical investigations would be required in search for materials showing this mechanism.

In conclusion, first-principles calculations are used to study the atomic and electronic structures of a 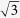-Ag surface in the presence of Au, Ag, and Na adatoms for different coverage levels. The adatoms tend to form clusters as the coverage level increases. The simulated ARPES spectra are consistent with the experimental spectra, and the *S*_1_ band shifts are reproduced well. The charge transfer calculations reveal that the Au and Na adatoms induce opposing charge transfer effects, and the charge transfer model and the rigid band model are not valid in this system. Instead, model calculations of the artificial structures and a simple tight-binding argument are applied to determine that the downward shift in the *S*_1_ band arise from strengthened substrate Ag-Ag bonds. The present findings describe a new class of mechanisms by which the electronic structures of a host material change upon introduction of impurity atoms. This mechanism provides a new framework for interpreting the electronic structures associated with the presence of impurity atoms in all kinds of the one-, two-, and three-dimentional systems.

## Methods

All calculations are performed using the Vienna *ab-initio* simulation package (VASP)^22^,^23^, which incorporates ultrasoft pseudopotentials^24^ and the general gradient approximation (GGA) of Perdew and Wang^25^ for the exchange-correlation energy of the electrons. We simulate the surfaces using a repeated slab model with a 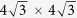 lateral periodicity to include six Si layers and 10 Å vacuum layers. The 2 × 2 k-point mesh, including the Γ point, is used for surface Brillouin integration. These parameters produce convergent results^20^. All atoms except for the bottom-most Si and H atoms are relaxed until the residual force acting on each atom drops below 0.02 eV/Å. The 13 Ry cutoff energy is used to expand the wave functions in the plane-wave basis in all calculations.

Charge transfer is calculated according to the Bader definition^26^, using the code implemented into VASP by Henkelman *et al.*^27^. In calculating the Bader charges, the projector-augmented waves^28^ are used instead of the ultrasoft pseudopotentials.

ARPES simulation has been performed by using the Fermi’s golden rule. The intensity of the ARPES simulation, *I*, is formulated by the equation^29^,^30^, 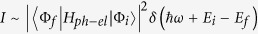, where the 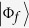, 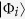, *H*_*ph*−*el*_, 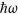, *E*_*i*_, and *E*_*f*_ are the Kohn-Sham final and initial states, the interaction Hamiltonian between the photon and the electron, the photon energy, and the initial-state and final-state eigenvalues, respectively. The unfolded energy band is obtained from the folded 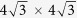 supercell eigenstates by investigating coefficients of redefined k-space vectors. We have implemented this formalism into the VASP code, independently of the recent work by Medeiros *et al.*^31^.

## Additional Information

**How to cite this article**: Kim, G. H. and Jeong, S. Change of Electronic Structures by Dopant-Induced Local Strain. *Sci. Rep.* **5**, 11227; doi: 10.1038/srep11227 (2015).

## Figures and Tables

**Table 1:** Calculated Bader charges of the most stable Au, Ag, and Na adatom structures on the 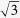-Ag surface.

**Structure**	**Adatom**	**Si trimers**	**Ag atoms**	**Substrate Si Atoms**
1Au	0.45	−0.29	−0.03	−0.13
1Ag	0.01	−0.01	0.09	−0.09
1Na	−0.80	0.42	0.44	−0.06

The Bader charges of the 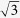-Ag substrate were set to zero. The minus sign indicates electron loss from the neutral atom.

## References

[b1] Sterm E. A. Rigid-band model of alloys. Phys. Rev. 157, 544 (1967).

[b2] Chen Y. L. *et al.* Experimental realization of a three-dimensional topological insulator, Bi_2_Te_3_. Science 325, 178 (2009). 19520912 10.1126/science.1173034

[b3] Zhang Y. *et al.* Doping effects of Sb and Pb in epitaxial topological insulator Bi_2_Se_3_ thin films: An *in situ* angle-resolved photoemission spectroscopy study. Appl. Phys. Lett. 97, 194102 (2010).

[b4] Gierz I., Riedl C., Starke U. R., Ast C. & Kern K. Atomic hole doping of graphene. Nano Lett. 8, 4603 (2008). 19053796 10.1021/nl802996s

[b5] HiraharaT., MatsudaI., UenoM. & HasegawaS. The effective mass of a free-electron-like surface state of the Si(111) 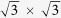 -Ag surface investigated by photoemission and scanning tunneling spectroscopies. Surf. Sci . 563, 191–198 (2004).

[b6] Pérez-DiesteV. *et al.* Orbital origin and matrix element effects in the Ag/Si(111)- 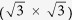 R30^°^ Fermi surface. Surf. Sci. 601, 742 (2007).

[b7] TongX., JiangC. S. & HasegawaS. Electronic structure of the Si(111) 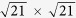 -(Ag+Au) surface. Phys. Rev. B 57, 9015 (1998).

[b8] CrainJ. N., GallagherM. C., McChesneyJ. L. & HimpselF. J. Doping of a surface band on Si(111) 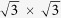 -Ag. Phys. Rev. B 72, 045312 (2005).

[b9] Liu C., Matsuda I., Hobara R. & Hasegawa S. Interaction between adatom-induced localized states and a quasi-two-dimensional electron gas. Phys. Rev. Lett. 96, 036803 (2006). 16486752 10.1103/PhysRevLett.96.036803

[b10] KonishiM. *et al.* 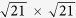 phase formed by Na adsorption on Si(111) 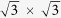 -Ag and its electronic structure. e-J. Surf. Sci. Nanotech. 3, 107–112 (2005).

[b11] JeongH., YeomH. W. & JeongS. Adatom-induced variations of the atomic and electronic structures of Si(111) 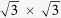 -Ag: A first-principles study. Phys. Rev. B 77, 235425 (2008).

[b12] AizawaH., TsukadaM. & Sato. First-principles study of Ag adatoms on the Si(111) 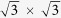 -Ag surface. Phys. Rev. B 59, 10923 (1999).

[b13] XieX. *et al.* Noble and alkali adatoms on a Si(111) 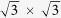 -Ag surface: a first-principles study. J. Phys. Condens. Matter 22, 085001 (2010).2138940310.1088/0953-8984/22/8/085001

[b14] Chaibi W., Peláez R. J., Blondel C., Drag C. & Delsart C. Effect of a magnetic field in photodetachment microscopy. Euro. Phys. J. D 58, 29–37 (2010).

[b15] Bilodeau R. C., Scheer M. & Haugen H. K. Infrared laser photodetachment of transition metal negative ions: studies on Cr^−^, Mo^−^, Cu^−^, and Ag^−^. J. Phys. B: At. Mol. Opt. Phys. 31, 3885 (1998).

[b16] Andersen T., Haugen H. K. & Hotop H. Binding Energies in Atomic Negative Ions: III J. Phys. Chem. Ref. Data 28, 1511 (1999).

[b17] Hotop H. & Lineberger W. C. Binding Energies in Atomic Negative Ions: II. J. Phys. Chem. Ref. Data 14, 731 (1985).

[b18] AizawaH., TsukadaM., SatoN. & HasegawaS. Asymmetric structure of the Si(111)- 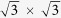 -Ag surface. Surf. Sci 429, L509–L514 (1999).

[b19] JeongH., YeomH. W. & JeongS. Immersion structures of monovalent metal adatoms on Ag/Si(111) 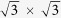 : First-principles study. Phys. Rev. B 76, 085423 (2007).

[b20] JeongS. & JeongH. Adatom-dependent diffusion mechanisms on a Ag/Si(111) 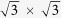 surface. Phy. Rev. B 81, 195429 (2010).

[b21] KimG. H. & JeongS. Adsorption and electronic structures of Na adatoms on the Ag/Si(111)- 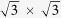 surface. J. Kor. Phys. Soc. 60, 1390 (2012).

[b22] Kresse G. & Hafner J. Ab initio molecular dynamics for liquid metals. Phys. Rev. B 47, R558 (1993). 10.1103/physrevb.47.558 10004490

[b23] Kresse G. & Furthmuller J. Efficient iterative schemes for ab initio total-energy calculations using a plane-wave basis set. Phys. Rev. B 54, 11169 (1997). 10.1103/physrevb.54.11169 9984901

[b24] Vanderbilt D. Soft self-consistent pseudopotentials in a generalized eigenvalue formalism. Phys Rev. B 41, R7892 (1990). 10.1103/physrevb.41.7892 9993096

[b25] Perdew J. P. & Wang Y. Accurate and simple analytic representation of the electron-gas correlation energy Phys. Rev. B 45, 13244 (1992). 10.1103/physrevb.45.13244 10001404

[b26] Bader R. Atomics in Molecules: A Quantum Theory, Oxford University Press, New York (1990).

[b27] Henkelman G., Arnaldsson A. & Jonsson H. A fast and robust algorithm for Bader decomposition of charge density. Comput. Mater. Sci. 36, 354 (2006).

[b28] Blöchl P. E. Projector augmented-wave method. Phys. Rev. B 50, 17953 (1994). 10.1103/physrevb.50.17953 9976227

[b29] Kim S., Ihm J., Choi H. J. & Son Y. W. Minimal single-particle Hamiltonian for charge carriers in epitaxial graphene on 4H-SiC(0001). *arXiv*: 0912.1210v1 (2009).

[b30] Ku W., Berlijin T. & Lee C.-C. Unfolding first-principles band structures. Phys. Rev. Lett. 104, 216401 (2010). 20867120 10.1103/PhysRevLett.104.216401

[b31] Medeiros P. V. C., Stafström S. & Björk J. Effects of extrinsic and intrinsic perturbations on the electronic structure of graphene: Retaining an effective primitive cell band structure by band unfolding. Phys. Rev. B 89, 041407 (2014).

